# A thermo-alkali stable and detergent compatible processive β-1,4-glucanase from Himalayan *Bacillus* sp. PCH94

**DOI:** 10.3389/fmicb.2022.1058249

**Published:** 2022-11-09

**Authors:** Vikas Thakur, Dharam Singh

**Affiliations:** ^1^Biotechnology Division, CSIR-Institute of Himalayan Bioresource Technology, Palampur, Himachal Pradesh, India; ^2^Academy of Scientific and Innovative Research (AcSIR), Ghaziabad, Uttar Pradesh, India

**Keywords:** glucanase, hydrolysis, thermostability, industrial applications, biorefinery

## Abstract

Present study reports a novel and robust GH9 processive endoglucanase β-1,4-glucanase from *Bacillus* sp. PCH94 (EGase^BL^) with thermo-alkali stable properties. The *EGase^BL^* gene was cloned in pET-28b(+) and expressed in *Escherichia coli* BL21(DE3) cells. The recombinant protein was purified 94-fold with a yield of 67.8%. The biochemical characterization revealed an active enzyme at a wide pH (4.0–10.0) and temperature (4–100°C). It showed a *Km* and *Vmax* of 1.10 mg/ml and 208.24 IU/mg, respectively, using β-glucan as a substrate. The EGase^BL^ showed dual activities for endoglucanase (134.17 IU/mg) and exoglucanase (28.76 IU/mg), assayed using substrates β-glucan and Avicel, respectively. The enzyme is highly stable in neutral and alkaline pH and showed a half-life of 11.29 h, and 8.31 h in pH 7.0 and 9.0, respectively. The enzyme is also compatible with commercial detergents (Tide, Surf, Ghadi, Raj, and Healing tree) of the Indian market and retained > 85% enzyme activity. Concisely, robustness, extreme functionality, and detergent compatibility endorse EGase^BL^ as a potential bioresource for the detergent industry, in addition to its implications for the bioethanol industry.

**Highlights**

– Cloning, expression, and purification of putative novel GH9 family β-1,4-glucanase.

– Processive endoglucanase with CBM3 domain and bi-functional (endo/exo) activity.

– Broad pH-temperature active and stable enzyme.

– Compatible with commercial detergent powders.

## Introduction

The global detergent market is growing at a CAGR of 6.3% after an estimated value of 154.34 billion in 2021.^1^ The detergent industry has experienced a revolution with the use of enzyme additives such as cellulase, amylase, and protease in detergents (Cavaco-Paulo et al., 1997; Niyonzima, 2019). These hydrolytic enzymes led to detergent formulations with efficient stain removal properties. Among them, alkaline cellulases have been the key additives in detergents for fabric softening and color brightness (Cavaco-Paulo, 1998; Niyonzima, 2019). In addition to the detergent industry, microbial enzymes are the hotspot to deal with a global challenge in alternate energy sources (Zhu et al., 2020; Antar et al., 2021). Among cellulases, the endoglucanase (EG; EC 3.2.1.4) is the key enzyme that carries out the breakdown of β-1,4-glycosidic bonds in cellulose chains. They belong to 12 glycosyl hydrolase (GH) superfamilies: 5, 6, 7, 8, 9, 12, 44, 45, 48, 51, 74, and 124 (Kim et al., 2016; Kumar and Naraian, 2018).

Endoglucanases (EGs) are required in various industries like biorefinery, paper, and pulp, and detergent for applications such as biomass conversion, biopulping, deinking, and biopolishing, respectively (Bhat, 2000; Belghith et al., 2001; Chandel et al., 2007, 2012; Kuhad et al., 2011; Kumar et al., 2018; Golgeri et al., 2022). The cellulolytic enzymes depolymerize the cellulosic biomass into simpler sugars, which are further fermented to produce bioethanol (Zhu et al., 2020). Also, in the detergent industry, cellulase assists in the softness, color brightness, and defibring of clothes (Miettinen-Oinonen and Suominen, 2002; Sadhu et al., 2013; Kumar and Naraian, 2018; Niyonzima, 2019). Nevertheless, the different industrial bioprocesses need endoglucanases to work under different conditions. For instance, the biorefinery needs cellulolytic enzymes to work at elevated temperatures and the pulp industry requires acidic cellulases. In contrast, the detergent industry requires an enzyme that works in alkaline environment (Kuhad et al., 2011; Ladeira et al., 2015; Bagewadi et al., 2016).

The endoglucanases act on their substrates through two action mechanisms, processive and non-processive. Most GH9 family EGs are processive, containing a catalytic domain (CD) and a carbohydrate-binding module (CBM; Konar et al., 2022). The CBMs are responsible for the processive nature of EGs (Chiriac et al., 2010). The processive endoglucanases (PEGs) show both endo- exo- bi-functional activities (Lv et al., 2021; Wu et al., 2022). It generates a higher diversity of hydrolysis products than exoglucanases (Wu and Wu, 2020). The first report of PEGs from *Thermomonospora fusca* was reported in 1993 (Irwin et al., 1993), followed by several others. The majority of PEGs studied so far belong to *Clostridium* sp., while *Paenibacillus*, *Bacillus*, *Cellulomonas*, *Ruminococcus*, and *Thermobifida* are among the other genera (Reverbel-Leroy et al., 1997; Wilson, 2004; Mejia-Castillo et al., 2008; Chiriac et al., 2010; Wu and Wu, 2020; Gavande et al., 2022). The bi-functional PEGs, combined with other cellulases, are more suitable for cellulose-based bioprocess development for bioethanol generation (Akram et al., 2018). The PEGs also have potential applications in deinking and biostoning (Wu et al., 2007; Liu et al., 2017).

PEGs are emerging as game-changing players in the cellulose-based industries. The lower stability and incompetence to work in extreme conditions is the biggest bottleneck of commercial cellulases (Han et al., 2020). Therefore, discovering robust cellulases compatible with multi-industrial applications is of prime necessity. Previously, we have isolated *Bacillus* sp. PCH94 from compost in the Western Himalayas and studied its biomass hydrolysis potential on damaged rice grain waste (Thakur et al., 2018, 2021). The current study investigated the biochemical characteristics of a recombinant processive EGase^BL^. Here, we subjected the purified EGase^BL^ enzyme to varied pH and temperature conditions to assess its compatibility with industrial bioprocess standards. The multi-dimensional properties of the enzyme are key stipulations for biorefinery, detergent, and textile industries.

## Materials and methods

### Bioinformatic analysis of β-1,4-glucanase sequence

The *Bacillus licheniformis* strain PCH94 was isolated in our lab and studied for its cellulolytic potential (Thakur et al., 2018, 2021). The gene *EGase^BL^* was obtained from a whole-genome sequence of *Bacillus* sp. PCH94 (Thakur et al., 2021) and submitted to the NCBI GenBank database with an accession number OM867537. The NCBI ORF finder program was used to identify the open reading frame in the gene sequence.^2^ The gene sequence was translated using the ExPASy translate tool.^3^ The protein sequence identity was confirmed by the NCBI protein BLAST (blastp) tool with the protein databank (PDB) database as a reference. The prediction of signal peptide in protein sequence was performed by SignalP server 5.0. The NCBI conserved domain database (CDD) was used to predict the conserved domains.^4^ The physicochemical properties like isoelectric point (pI) and molecular weight (MW) of the protein sequence of EGase^BL^ were identified using ProtParam.^5^ The phylogenetic tree was constructed to study the evolutionary relationship of EGase^BL^ protein sequence through Mega 7.0. The protein structure analysis was carried out using the RaptorX Property web server (Wang et al., 2016). The homology model of EGase^BL^ protein was generated using the SWISS-MODEL server (Waterhouse et al., 2018).

### Cloning and heterologous expression of EGase^BL^ in pET-28b(+)/*Escherichia coli* BL21(DE3)

The *EGase^BL^* gene was amplified from genomic DNA using gene-specific primers, inserted with restriction enzymes sites (indicated with underline) for NcoI (Forward primer 5′-CATG CCATGG GC ATG AAA GCG CTT TGT TTG GC-3′) and XhoI (Reverse primer 5′-CCG CTCGAG GTA ACC GGG CTC ATG TCC GAA-3′). The PCR to amplify the gene was carried out under the following conditions: initial denaturation at 95°C for 2 min; 35 cycles of 95°C for 30s; 58°C for 30s; 72°C for 2 min; and a final extension at 72°C for 7 min. The amplified gene product was purified using the FavorPrep PCR purification kit (FAVORGEN Biotech Corp, Taiwan) as per the manufacturer’s instructions. The final *EGase^BL^* gene product and pET-28b(+) vector were further subjected to double restriction digestion with Fast digest NcoI and XhoI restriction enzymes. The digested products were purified and ligated using T4 DNA ligase. The ligated product was transformed into competent BL21 (DE3) cells using the heat-shock method. The colonies were isolated on LB plates with 50 μg/ml kanamycin, and positive transformants were screened through the colony PCR technique. The desired cloned gene in positive transformants was also confirmed through T7 promoter-based sequencing in ABI PRISM™ 3,130 × l Genetic Analyzer (Applied Biosystems, United States).

The expression of *EGase^BL^* gene was carried out under optimized conditions, i.e., 1.0 ml of overnight grown seed culture was inoculated in a 50 ml LB broth containing Kanamycin (50 μg/ml) and incubated at 37°C/200 rpm. The culture was grown until OD_600_ reached 0.4–0.6, supplemented with 0.1 mM Isopropyl β-D-1-Thiogalactopyranoside (IPTG), and transferred to 28°C/200 rpm for 24 h. The cells were harvested, centrifuged, and the pellet was re-suspended in lysis buffer containing Tris–HCl buffer (25 mM/pH 8.0), 300 mM of NaCl, and 5 mM imidazole. The cells were homogenized using an ultrasonic sonicator (Ultrasonic Processor SKL-150D) with 10s on/off, 5 cycles. After sonication, the lysed cells solution was centrifuged at 12000 *g* for 10 min at 4°C, and cell-free supernatant (CFS) was used as a crude enzyme for further studies. The expression of the desired protein was visualized on a 10% SDS-PAGE.

### Enzyme activity assay

The enzyme activity of the EGase^BL^ enzyme was determined using the DNSA method for reducing sugars (Miller, 1959). The reaction conditions were as follows: incubation temperature: 50°C, time: 60 min, pH: 8.0/25 mM, and substrate: 1.0% (w/v) barley β-glucan (prepared in 25 mM pH 8.0 Tris–HCl buffer), enzyme: substrate ratio-1:2 (*v*/*v*). The enzyme-specific activity of β-1,4-glucanase was defined as μ moles of reducing sugar released from β-glucan per minute per mg of the enzyme under the assay conditions. Each reaction with its controls was carried out in triplicates in all the experiments. The observed values were subjected to standard deviation (SD), and ± 2 SD was considered significant.

### Purification of recombinant *EGase^BL^* and its proteomic analysis

The EGase^BL^ protein was expressed and produced in 1.0 liter LB broth media. The HisPur Cobalt superflow resin was used for the purification of recombinant EGase^BL^. The chromatography column was equilibrated using a basic buffer containing 25 mM Tris–HCl pH 8.0, 300 mM NaCl, and 5 mM imidazole. The CFS was filtered through a 0.45 μM syringe filter and loaded on the pre-equilibrated column. The CFS was further subjected to IMAC (Immobilized metal affinity chromatography) resin-based purification using HisPur™ cobalt Superflow Agarose. The CFS was allowed to pass through the gravity column twice for the maximum binding. The column was washed twice with the basic buffer using 10 column volumes to remove non-specific proteins. The desired EGase^BL^ protein bound to the resin was eluted by using elution buffer, i.e., Tris–HCl pH 8.0, 300 mM NaCl, and 150 mM imidazole. The total amount of protein was estimated by using the Bradford assay (Bradford, 1976). The purity of the eluted fraction was visualized on a 10% SDS-PAGE gel.

### Biochemical characterization of purified *EGase^BL^*

The one factor at a time (OFAT) scheme was used to study the biochemical characteristics of purified EGase^BL^ and obtain its highest activity. The reaction pH range was calculated using varied buffer systems consisting of sodium citrate (pH 3.0–5.0), potassium phosphate (pH 6.0–7.0), Tris–HCl (pH 8.0–10.0), and sodium carbonate–bicarbonate (pH 9.0–10.0). The reaction conditions were as follows: temperature: 50°C, time: 60 min, and substrate: 1.0% (w/v) barley β-glucan. The buffer system with the highest specific activity was considered the optimum pH for the reaction. Furthermore, the optimum reaction temperature was assessed under optimized pH in 60 min, and 1.0% (w/v) barley β-glucan substrate. The reactions were incubated at a temperature range of 4–100°C. The enzyme concentration in the reaction was optimized under optimum pH and temperature conditions. The enzyme concentration was varied from 0.1 to 5.0 μg per reaction and incubated for 30 min. Finally, the incubation time was optimized from a varying range from 5 to 30 min under previously optimized conditions.

### Assessment of kinetic parameters of purified *EGase^BL^*

The kinetic parameters like Michaelis constant (*K*_m_) and maximum reaction velocity (*V*_max_) were estimated on EGase^BL^ activity on β-glucan under optimized reaction conditions. The reactions were carried out at 60°C/ pH 7.0 buffer (25 mM Potassium Phosphate). The substrate concentration varied from 0.05 to 0.5% to a constant enzyme concentration. The Michaelis–Menten equation and double-reciprocal Lineweaver–Burk plots were used for calculating *K*_m_ and *V*_max_. The multi-substrate-specificity of EGase^BL^ was also studied on β-glucan, Avicel, filter paper, cellobiose, beechwood xylan, and starch.

### pH and thermal stability of purified *EGase^BL^*

The purified enzyme was incubated in various buffer systems for 5 h to investigate the effect of pH on enzyme activity. The three different buffers (25 mM), i.e., Citrate buffer pH 5.0, Potassium Phosphate buffer pH 7.0, and sodium carbonate–bicarbonate pH 9.0, were used. The enzyme reaction was performed every hour, and the half-life (*t*_1/2_) of the enzyme at different pH levels was calculated. Similarly, to study the half-life of EGase^BL^ at different temperatures, the enzyme was incubated at 50, 60, 70, and 80°C.

### Detergent stability of purified *EGase^BL^* in commercial detergents

The stability of purified EGase^BL^ was studied in various domestic Indian detergent powders such as Tide, Surf, Ghadi, Raj, and Healing tree. The detergent solution concentration was kept at 1.0% to stimulate washing conditions. Before enzyme addition, detergents were pre-incubated in boiling water for 10 min to deactivate existing enzymes (Niyonzima, 2019). The enzyme was incubated in a 1.0% detergent solutions for 30 min at room temperature. The enzyme reaction was performed following the incubation, and residual enzyme activity was calculated.

## Results and discussion

### Genome mining and bioinformatic analysis of *EGase^BL^* gene

**Figure 1:**
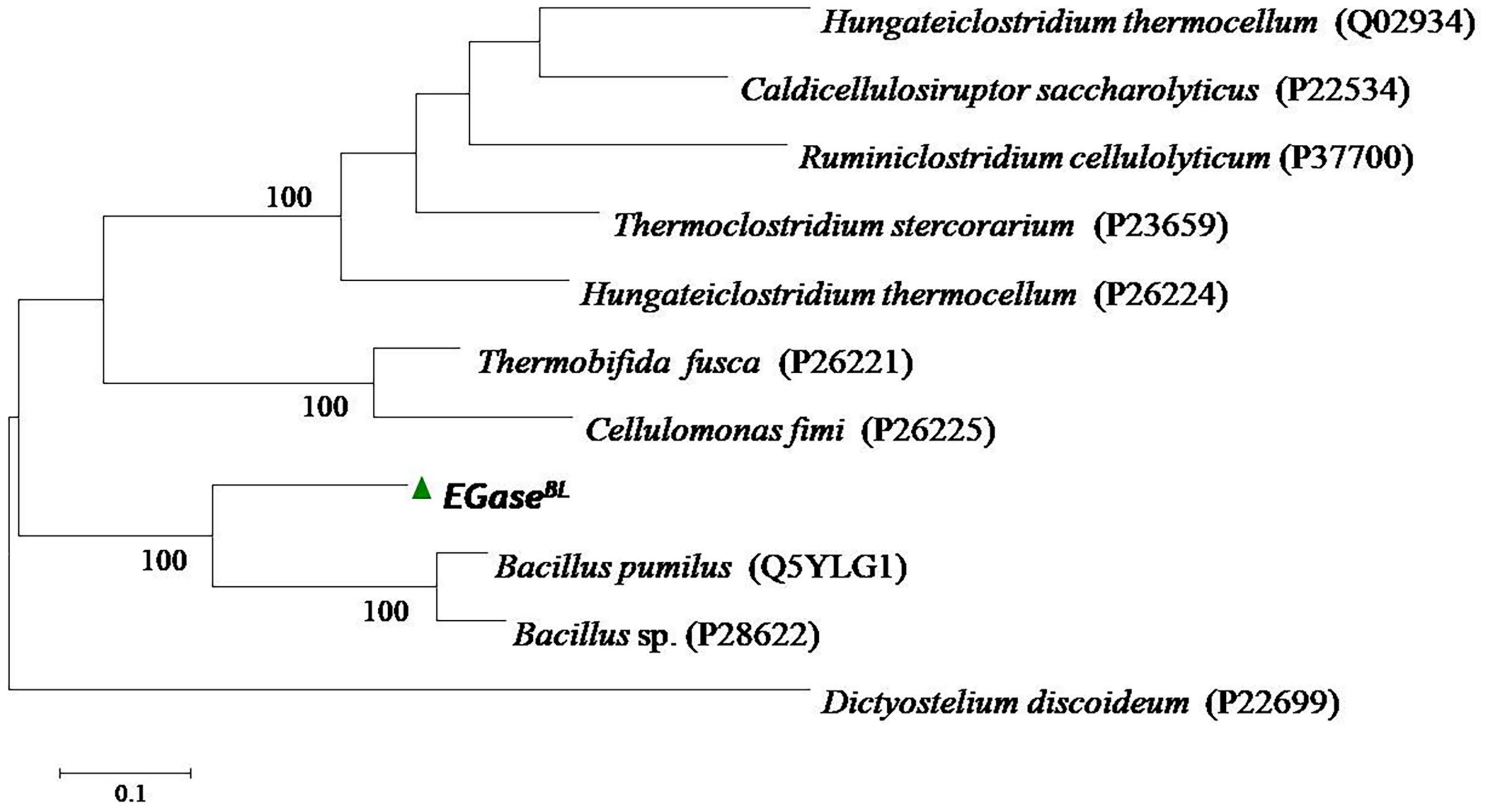
The phylogenetic analysis of the protein sequence of β-1,4-glucanase (EGase^BL^) of *Bacillus* sp. PCH94 with the closest matches in the Protein Data Bank (PDB) database.

**Figure 2:**
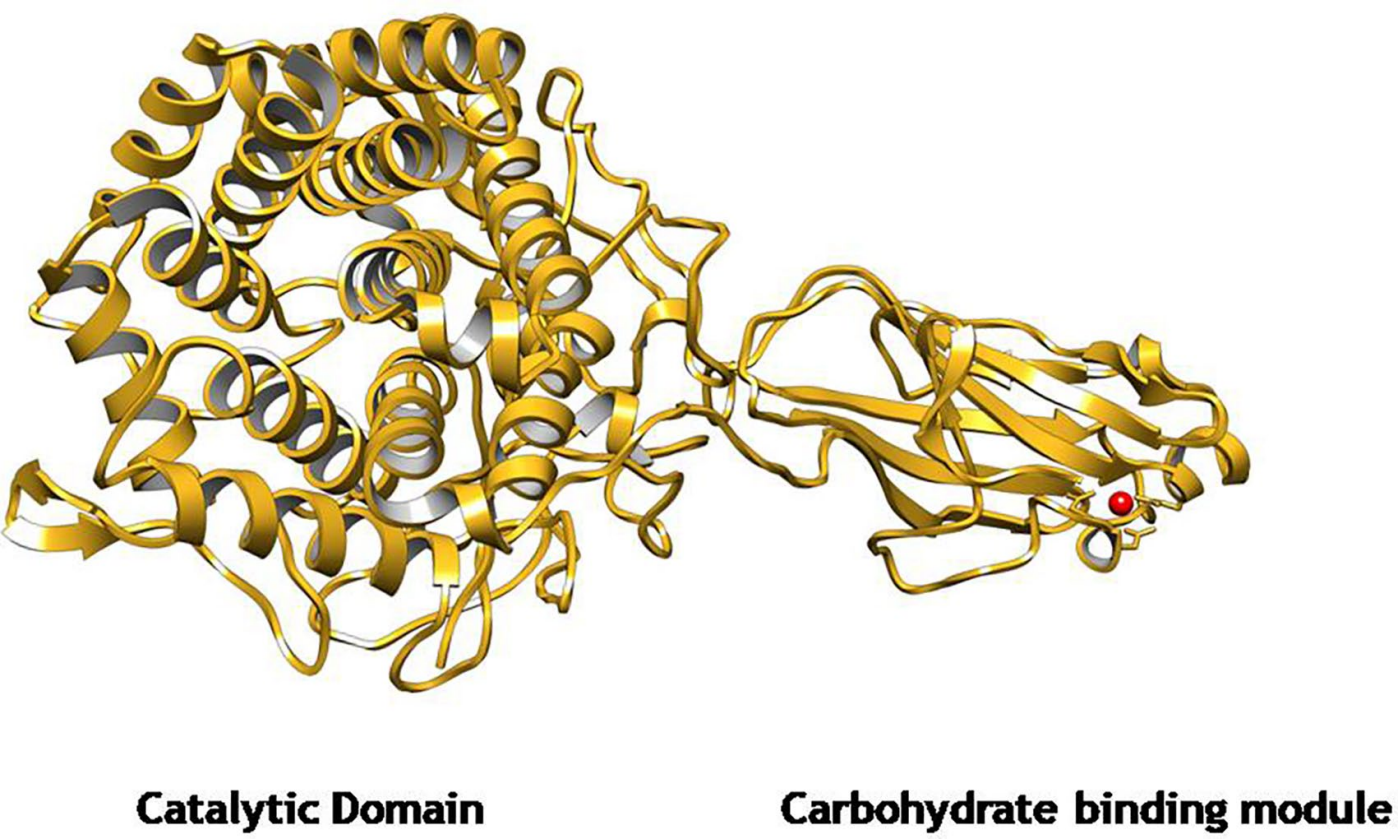
The predicted homology model of GH9 family β-1,4-glucanase (EGase^BL^) using Endoglucanase 9G from *Clostridium cellulolyticum* as a template in SWISS-MODEL server. The structure depicts a catalytic domain (CD) and a carbohydrate-binding module (CBM3).

**Figure 3:**
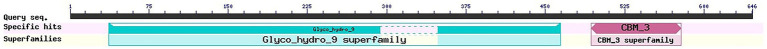
The conserved domain prediction in the protein sequence of GH9 β-1,4-glucanase using the NCBI database.

The gene *EGase^BL^* consisted of 1,941 bp and was mined from the whole-genome sequence of *Bacillus* sp. PCH94 (Thakur et al., 2021). The gene’s nucleotide sequence was submitted to NCBI GenBank with the accession number OM867537. The ExPASy translate server revealed that EGase^BL^ protein comprises 647 amino acids. The NCBI protein blast showed 53.08% similarity in the PDB database. The phylogenetic analysis of the EGase^BL^ protein sequence with the closest matches in the PDB server was carried out. The EGase^BL^ made a separate clade to its closest matches with protein sequences of *Bacillus pumilus* and *Bacillus* sp. (Figure 1). The percentage (%) similarity and phylogenetic analysis of the protein sequence of EGase^BL^ endorse it to be a putative novel protein. It has a molecular weight of 72.6 kDa and a theoretical pI of 5.34. The sequence contains 83 negatively and 61 positively charged residues. The instability index (II) is 28.88, classifying this protein as stable. The protein is extracellular in nature, as predicted by SignalP server. Further protein sequence analysis revealed that the protein belongs to the Glycosyl hydrolase-9 (GH9) family as predicted from the SWISS-MODEL server (Figure 2). Also, it contains a carbohydrate-binding module, i.e., CBM3 (Figure 3). In fact, the presence of CBM along with catalytic domains is a key feature of PEGs. The PEGs act as traditional EGs without CBM3 (Kumar et al., 2019). Further, the protein sequence analysis performed by the RaptorX Property web server predicted 34% alpha-helix, 13% beta-sheet, and 52% coil regions (Supplementary Figure S1).

### Cloning, heterologous expression, and purification of gene encoding β-1,4-glucanase

**Figure 4:**
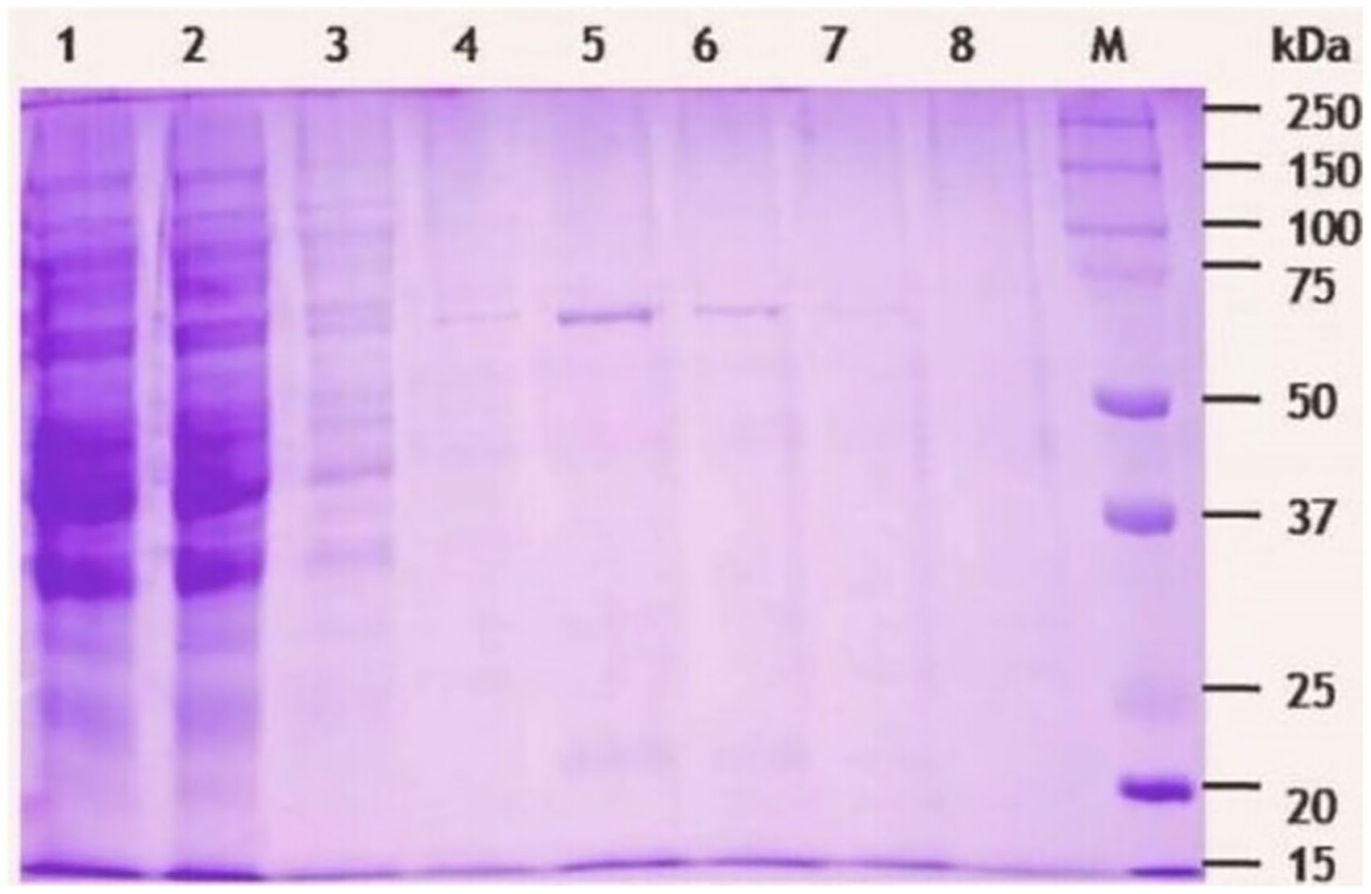
SDS-PAGE (10%) analysis of β-1,4-glucanase (EGase^BL^) purified using HisPur Cobalt superflow resin. The lane 1 to 9 represents total lysate, flow through, wash, elute 1(10 mM imidazole), elute 2 (50 mM imidazole), elute 3 (100 mM imidazole), elute 4 (150 mM imidazole), blank, and protein marker, respectively.

The β-1,4-glucanase (*EGase^BL^*) gene was amplified from the genomic DNA of *Bacillus* sp. PCH94 using gene-specific primers. The pET-28b(+)-EGase^BL^ construct was transformed in the expression host *E. coli* BL21(DE3) (Supplementary Figure S2). The positive transformants with the target gene were induced with 0.1 mM IPTG in LB media at 37°C and expressed under optimized conditions, i.e., 200 rpm/ 28°C. The maximum expression of EGase^BL^ was observed after 36 h at 28°C. The cell-free supernatant’s (CFS’s) quantitative estimation of β-1,4-glucanase activity showed the specific activity of 0.205 IU/mg on the β-glucan substrate. The use of affinity protein purification resulted in a single-step 94-fold purification of protein with a yield of 67.8%. Under unoptimized enzyme reaction conditions, the purified protein showed 19.35 IU/mg specific activity (Table 1). The purified protein was visualized on 10% SDS-PAGE, which revealed a distinct ~ 72 kDa band of purified EGase^BL^ (Figure 4).

**Table 1:** Protein purification of recombinant β-1,4-glucanase using HisPur Cobalt superflow resin.

Sample	Total protein (mg)	Specific activity (IU/mg)	Total units (U)	Yield (%)	Purification fold
Crude	285	0.205	58.42	100	1
Purified	2.05	19.35	39.66	67.88	94

Previously, the EGs belonging to different GH families have been cloned from various sources. Earlier, 63.5 and 64 kDa GH5 recombinant endoglucanases from *Paenibacillus* sp. were purified using the Ni-NTA column, resulting in 22.33 and 44.9% yields with activities of 7.55 and 3.63 IU/mg, respectively (Dhar et al., 2015a,b). A 41.56 kDa GH8 EGs was purified using High-Q and CHT-II column chromatography revealed 2.2 IU/mg activity with 15.9% recovery (Na et al., 2015). Similarly, a 59 kDa GH5 family recombinant EG from *Bacillus subtilis* was purified using the Ni-NTA column, giving the highest activity of 7.65 IU/mg (Guan et al., 2017). The purification scheme used in this study for EGase^BL^ has resulted in a higher enzyme recovery, purification fold, and specific activity than in previous studies. The highly purified enzyme can lead to efficient substrate conversion with high specificity.

### Biochemical characterization of purified *EGase^BL^*

**Figure 5:**
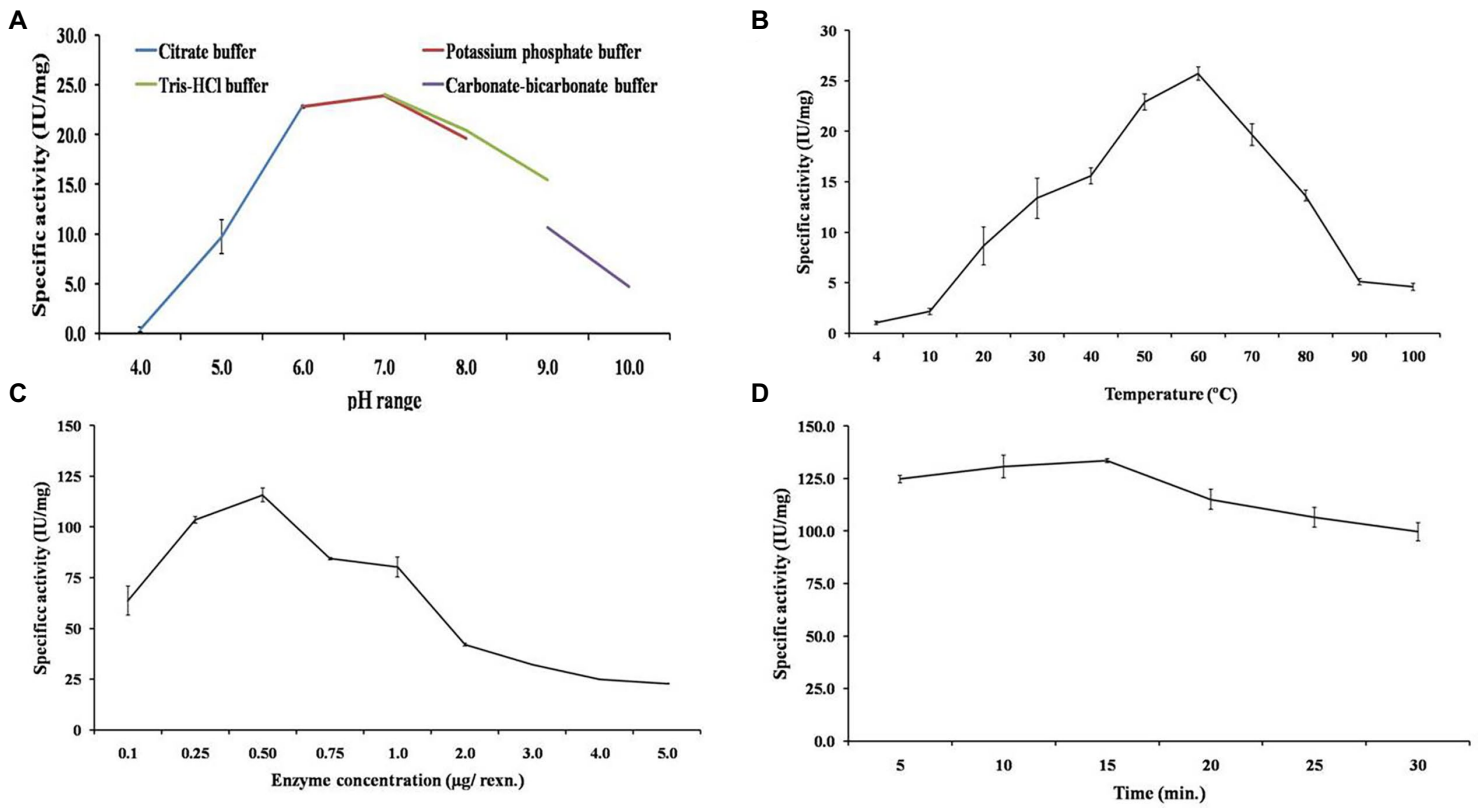
Biochemical characterization of purified β-1,4-glucanase (EGase^BL^) to obtain the optimum enzymatic conditions. The various parameters such as **(A)** optimum pH, **(B)** optimum temperature, **(C)** enzyme concentration, and **(D)** incubation time, were shown.

The buffer pH is a key parameter for optimum activity of enzyme. So, the enzyme activity was examined from pH 4.0 to 10.0. The highest specific activity of 24.06 IU/mg was obtained at pH 7.0 in 25 mM Tris–HCl buffer (Figure 5A). The relative activity of 70 and 64% was observed at pH 5.0 and 9.0, respectively. The activity decreased to ~0.8 and 19% at pH 4.0 and 10.0, respectively. Furthermore, the effect of temperature on EGase^BL^ activity was studied in a varied temperature range of 4–100°C, with the highest activity of 25.72 IU/mg observed at 60°C (Figure 5B). Relative activity of more than 50% was observed at a temperature range of 30–80°C. Also, 3.9 and 17.8% of relative activity was observed at 4°C and 100°C, respectively. The enzyme load plays a significant role in converting substrate into products. Hence, the effect of varied enzyme concentrations in the reaction was studied. The enzyme concentrations varied from 0.1 to 5.0 μg, and 0.5 μg per reaction was observed as the optimum enzyme concentration to achieve the highest specific activity of 115.7 IU/mg (Figure 5C). Further, the initial reaction rate was studied by optimizing the incubation time of the reaction. The incubation time of 10 to 30 min with 10 min intervals was studied. After 10 min of incubation, the specific activity of 130.6 IU/mg was achieved (Figure 5D). Concisely, biochemical characterization led to optimized reaction conditions, which are as follows: pH 7.0 Tris–HCl buffer, temperature 60°C, enzyme concentration 0.5 μg, and reaction time 10 min. Similarly, there are reports for the recombinant EG with a pH range of 4.0 to 9.0 and a temperature range of 20–70°C (Furtado et al., 2011). The rumen metagenome-derived EG showed a pH range of 4.0–10.0 and a temperature range of 20–70°C (Song et al., 2017). The EG from *Thermotoga maritima* showed a pH range of 4.0–8.0 (optimum pH 5.0) and a temperature range of 30–100°C (optimum temperature 95°C; Wang et al., 2020). The EGase^BL^ in the present study has shown a broader window of pH and temperature than the earlier reported studies. Such wider pH and temperature range has definitive advantages in the varied industrial bioprocesses.

### Investigation of kinetic parameters and substrate-specificity

**Figure 6:**
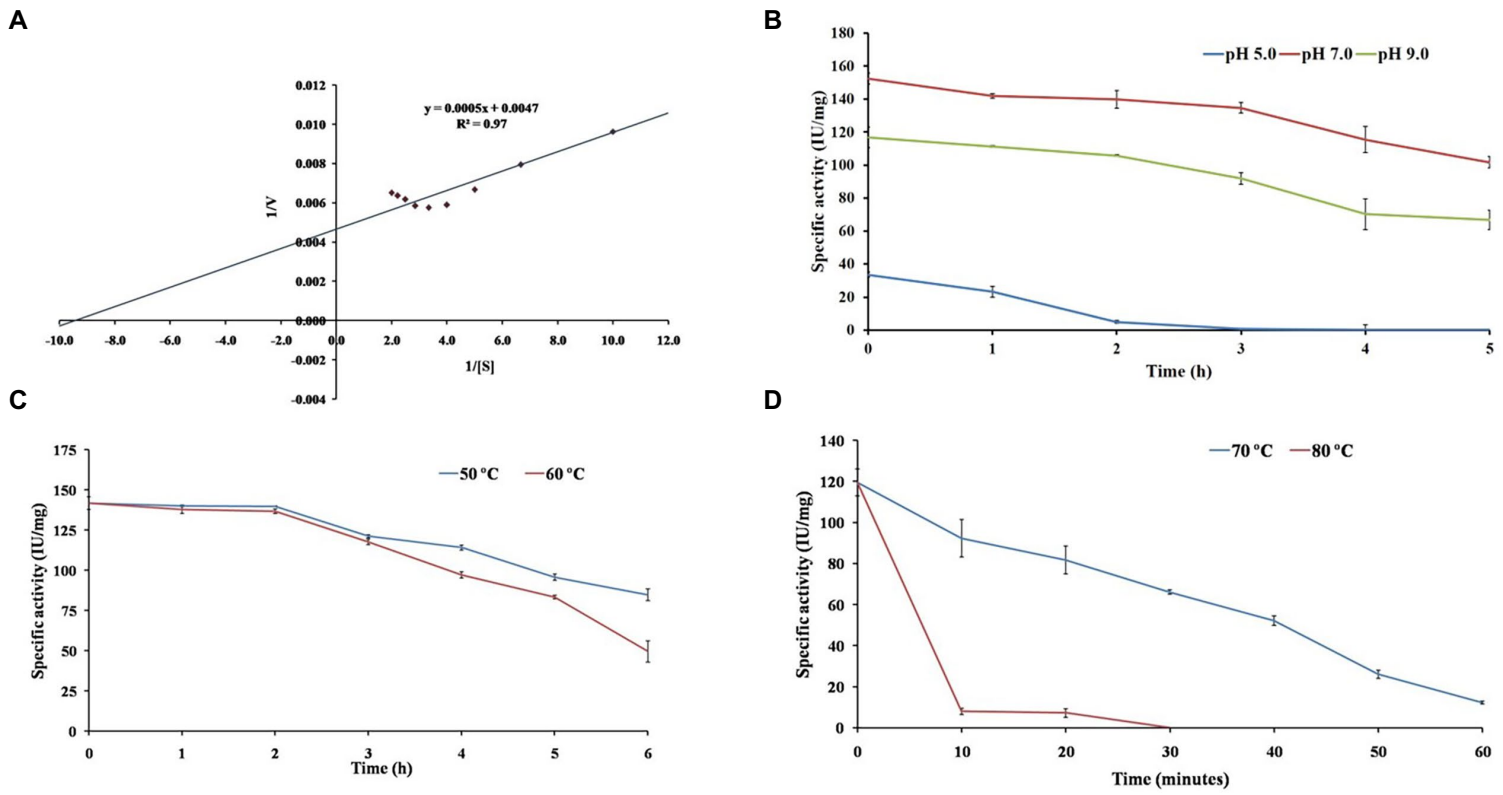
The figure depicts investigating β-1,4-glucanase (EGase^BL^) for **(A)** enzyme kinetics on β-glucan substrate, **(B)** pH stability at pH 5.0, 7.0 and 9.0, **(C)** thermal stability at 50 and 60°C, and **(D)** thermal stability at 70 and 80°C.

The Lineweaver–Burk double-reciprocal plot revealed that the EGase^BL^ follows Michaelis–Menten kinetics for β-glucan (Figure 6A). The *K*_m_ and *V*_max_ values of purified EGase^BL^ were 1.10 mg/ml and 208.24 IU/mg, respectively. The EG of *Raoultella ornithinolytica* showed *K*_m_ of 8.6 mg/ml and *V*_max_ of 74.9 IU/mg for β-glucan (Scapin et al., 2017). The recombinant EG of *Enterobacter* sp. had *K*_m_ and *V*_max_ of 13.5 mg/ml and 109.6 IU/mg, respectively, on β-glucan (Ontañon et al., 2019). A GH8 EG from *Bacillus subtilis* was estimated for *K*_m_ (1.78 mg/ml) and *V*_max_ (50.09 IU/mg) on CMC as a substrate (Huang et al., 2021). The EGase^BL^ has shown low *K*_m_ and high *V*_max_ values compared to these recent studies. The lower *K*_m_ suggests a high affinity for β-glucan, representing that it would take less substrate to reach *V*_max_.

The substrate-specificity of purified EGase^BL^ was studied on polysaccharides like β-glucan, Avicel, beechwood xylan, starch, cellobiose, and filter paper. The enzyme showed a specific activity of 134.17 IU/mg and 28.76 IU/mg on β-glucan and Avicel, respectively. While, with the rest of the other substrates, no enzymatic activity could be detected. The results imply that the enzyme has dual endoglucanase and avicelase/exoglucanase activity. The bi-functional activity profile and presence of CBM3 confirm the processive nature of EGase^BL^.

### pH and thermostability profile of purified *EGase^BL^*

The EGase^BL^ has shown wide pH and temperature working range. Therefore, the prolonged effect of pH and temperature on the enzyme was studied. The half-life (t_1/2_) of 0.80, 11.29, and 8.31 h was observed at pH of 5.0, 7.0, and 9.0 (Figure 6B). The enzyme could retain 90% residual activity after 1.0 h of incubation at pH 7.0 and 9.0. Interestingly, more than 75, and 60% activity at pH 7.0 and 9.0, respectively, were retained even after 5.0 h of incubation. The current results suggested that enzyme has the highest stability at neutral pH and is more stable at alkaline than acidic pH. The stability of EGase^BL^ in an alkaline environment is suitable for application as an additive in the detergent industry.

In addition to pH, thermal stability is a key factor for EGs, especially for their application in biomass saccharification. Therefore, thermal stability was evaluated by incubating the purified EGase^BL^ at 50, 60, 70, and 80°C. The half-life (t_1/2_) of 14.41 h, 8.63 h, 27 min, and > 5 min was observed at 50, 60, 70, and 80°C, respectively (Figures 6C,D). The enzyme showed optimum activity at 60°C and retained > 80% residual activity after 4.0 h of incubation. Previously, a β-1,4-glucanase from *Paenibacillus* sp. revealed a half-life of 60 min at 60°C (Na et al., 2015). The half-life of 3.0 h at 50°C was reported from the EG of *Raoultella ornithinolytica* (Scapin et al., 2017). The glucanase from *Bacillus* sp. lost 50% residual activity after incubation at 60°C for 10 min (Jang et al., 2021). Comparatively, the EGase^BL^ has shown much higher stability than the β-1,4-glucanases discussed above. Thermostability enables the enzyme to hydrolyze at a higher temperature more efficiently. Hence, it lowers the risk of contamination compared to mesophilic enzymes in a process (Akram et al., 2018).

### Detergent compatibility of purified *EGase^BL^*

**Figure 7:**
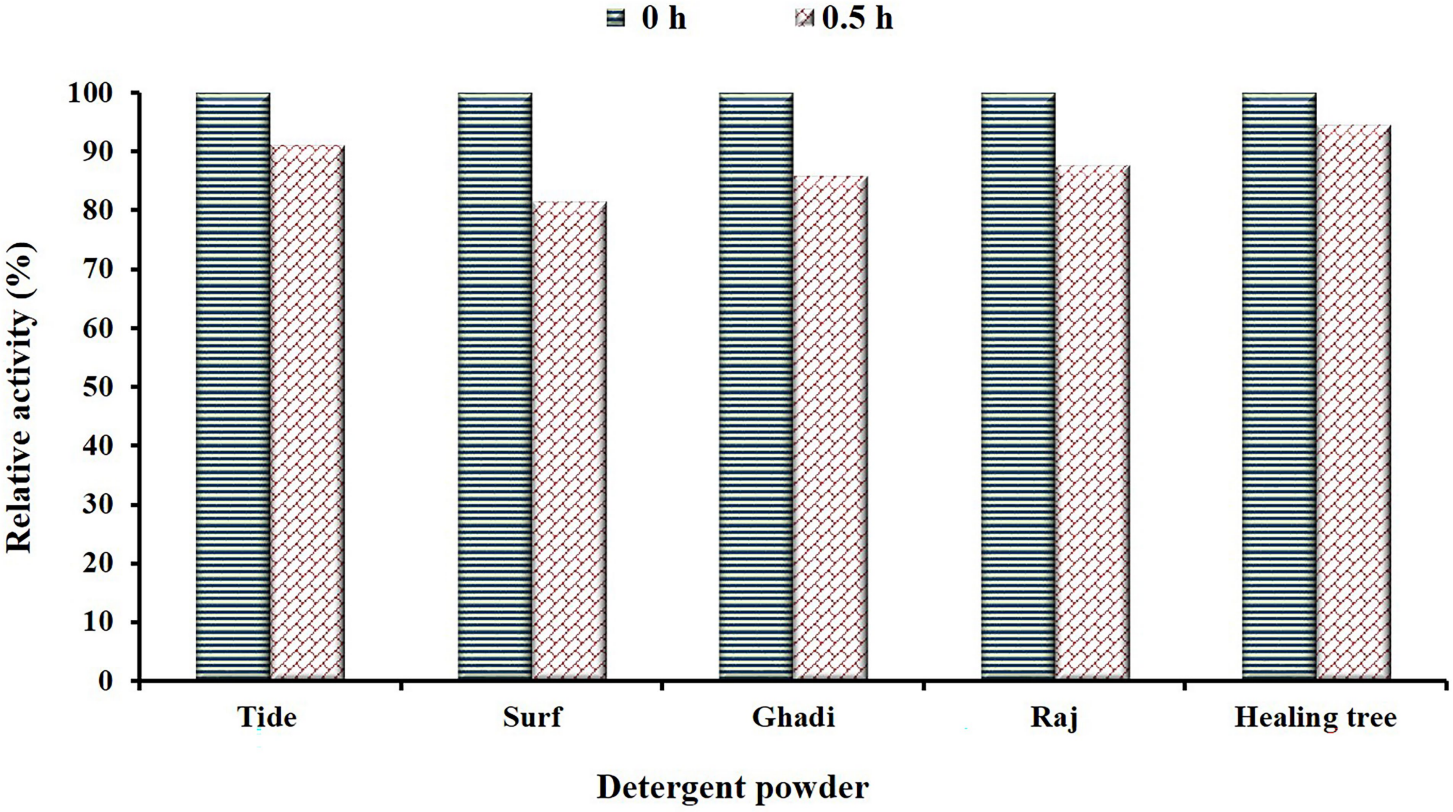
Depicts the stability/compatibility of β-1,4-glucanase (EGase^BL^) in commercial detergents (Tide, Surf, Ghadi, Raj, and Healing tree) available in the local market. The enzyme was incubated for 30 min in detergents at a room temperature condition.

The EGs are key additives to the enzyme cocktails used in the detergent powders. Most detergent brands instruct to keep clothes pre-soaked in detergent water for 20–30 min before washing. Therefore, we studied the compatibility/stability of purified EGase^BL^ by incubating it for 30 min in detergent powders available in the local market. The enzyme retained 91.1, 81.5, 85.9, 87.7, and 94.6% activity in Tide, Surf, Ghadi, Raj, and Healing tree, respectively (Figure 7). Previously, the residual activity of 72, 65, and 57% were reported for Ariel, Surf Excel, and Tide; respectively (Sadhu et al., 2013). Thermo-tolerant endoglucanase showed 50% residual activity in Surf Excel, Tide, Ariel, Wheel, and Patanjali detergent powders after 1.0 h (Joshi et al., 2021). The higher stability of EGase^BL^ in commercial detergents suggested it as a potential additive in the detergent industry.

## Conclusion

A novel thermo-alkali stable and detergent compatible β-1,4-glucanase (EGase^BL^) was cloned, expressed, and purified. The sequence analysis, presence of CBM module, and bi-functional activity confirm the processive nature of the GH9 family EGase^BL^. The recombinant protein was highly purified to 94 fold with a recovery of 67.7%. Also, the physicochemical characterization revealed the robust properties of EGase^BL^ activity and stability in broad pH and temperature ranges. The enzyme revealed remarkable stability (> 85%) in commercial detergents for 30 min. The compatibility and stability of EGase^BL^ in commercial detergents are promising for the detergent industry. In addition, the kinetics analysis showed a low *K*_m_ (1.10 mg/ml) and high *V*_max_ (208.6 IU/mg) on the β-glucan substrate. The ability of EGase^BL^ for β-glucan and Avicel hydrolysis and broad pH/temperature stability makes it too a potential player in the biorefinery industry. Therefore, the recombinant EGase^BL^ has shown promise for varied industrial applications, and its future explorations could lead to astonishing bioprocess developments.

## Data availability statement

The datasets presented in this study can be found in online repositories. The names of the repository/repositories and accession number(s) can be found in the article/Supplementary material.

## Ethics statement

The authors declare that this research did not involve human participants and/or animals. The authors declare that this work involved no unusual hazard, animal, or human subjects.

## Author contributions

VT: designed, performed experiments, data analysis, and manuscript writing. DS: conceived the study, research methodology and design, data interpretation, manuscript writing, and research supervision. All authors contributed to the article and approved the submitted version.

## Funding

VT duly received support from the Senior Research Fellowship (award no. 31/54(0144)/2019-EMR-1) from the Council of Scientific and Industrial Research (CSIR), New Delhi, India. DS received financial support from CSIR (grant MLP0143), New Delhi, under Niche Creating Project and Department of Biotechnology, New Delhi, in the form of GAP0130 under “Himalayan Bioresources Mission.”

## Conflict of interest

The authors declare that the research was conducted in the absence of any commercial or financial relationships that could be construed as a potential conflict of interest.

## Publisher’s note

All claims expressed in this article are solely those of the authors and do not necessarily represent those of their affiliated organizations, or those of the publisher, the editors and the reviewers. Any product that may be evaluated in this article, or claim that may be made by its manufacturer, is not guaranteed or endorsed by the publisher.

## Footnotes

^1^ https://www.polarismarketresearch.com/industry-analysis/laundry-detergent-market

^2^ https://www.ncbi.nlm.nih.gov/orffinder/

^3^ https://web.expasy.org/translate/

^4^ https://www.ncbi.nlm.nih.gov/Structure/cdd/docs/cdd_search.html

^5^ https://web.expasy.org/protparam/
