# Supplementary material for: A thermo-alkali stable and detergent compatible processive β-1,4-glucanase from Himalayan Bacillus sp. PCH94
Source: Front Microbiol. 2022 Nov 9;13:1058249. doi: 10.3389/fmicb.2022.1058249 (PMC9682278; doi:10.3389/fmicb.2022.1058249)

**Supplementary figure**

**Fig.S1** The protein sequence based secondary structure analysis of β-1,4-glucanase using the RaptorX Property web server.


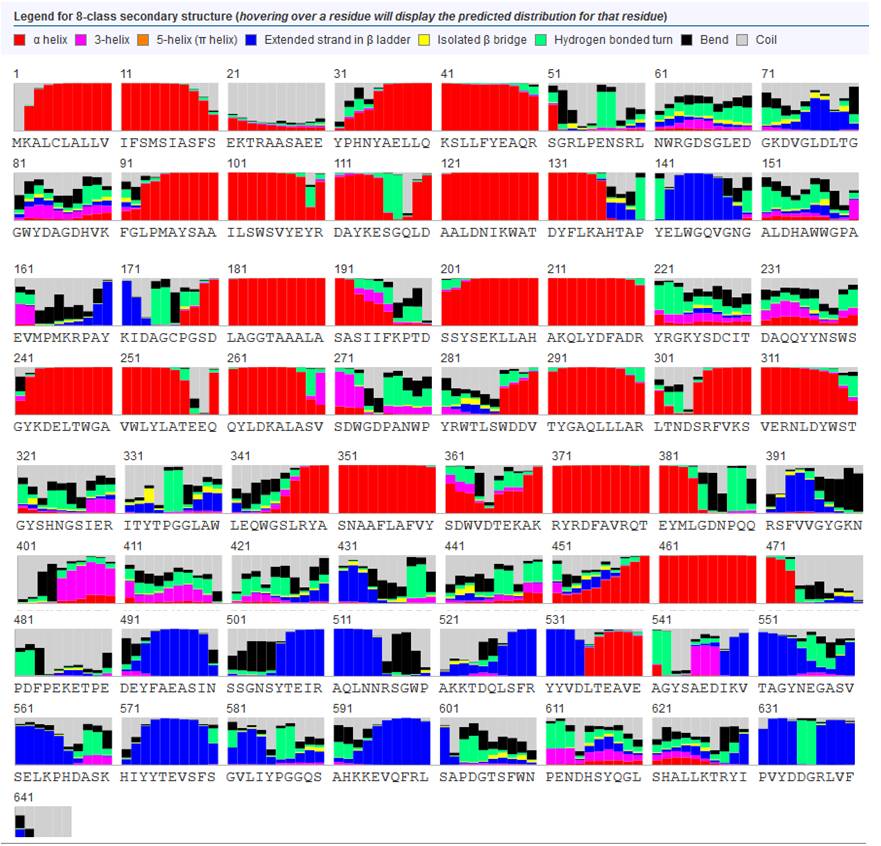


**FigS2** The figure represents the scheme used for cloning β-1,4-glucanase gene in pET 28a(+) vector.


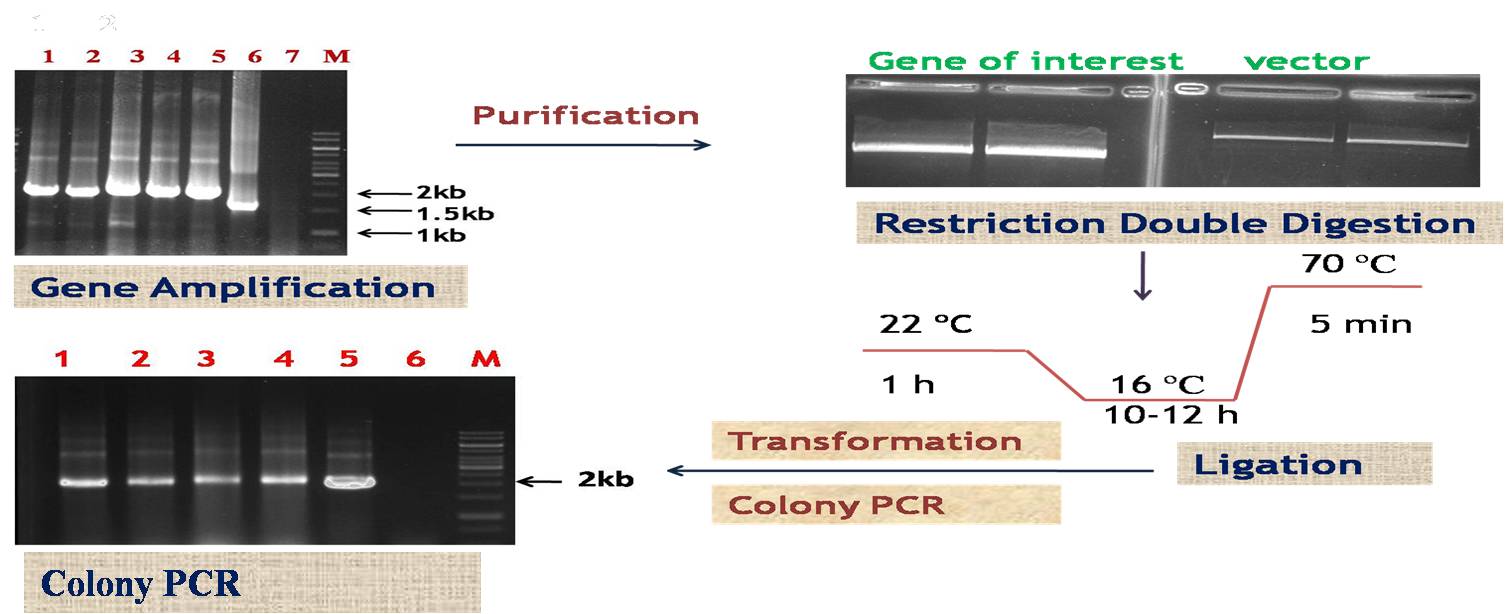

Supplement: Supplementary file 1 [file Data_Sheet_1.docx]
