# Supplementary figures and images for: A thermo-alkali stable and detergent compatible processive β-1,4-glucanase from Himalayan Bacillus sp. PCH94
Source: Front Microbiol. 2022 Nov 9;13:1058249. doi: 10.3389/fmicb.2022.1058249 (PMC9682278; doi:10.3389/fmicb.2022.1058249)

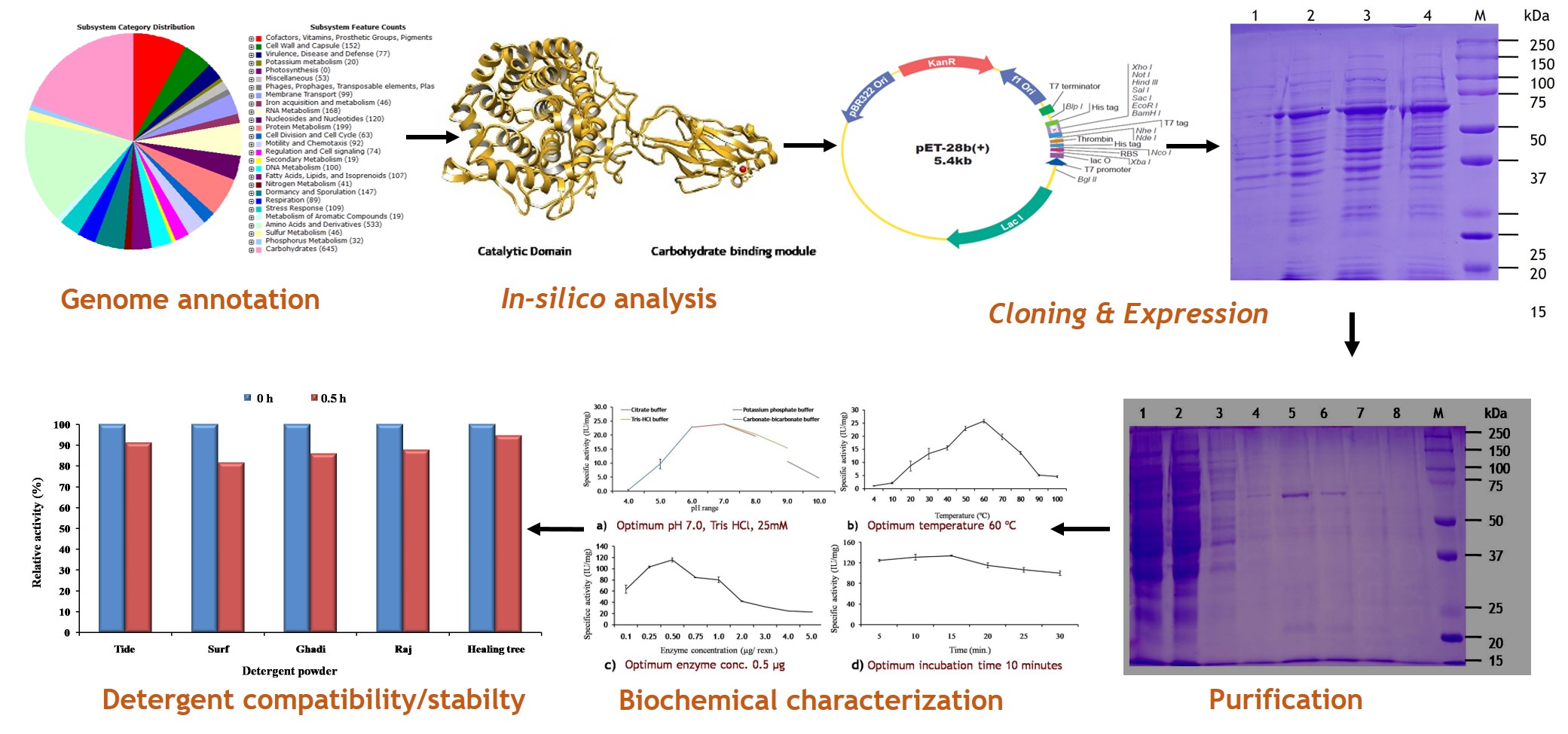

Supplement: Supplementary file 2 [file Image_1.JPEG]
